# Internet-based cognitive behavioral therapy for improving health-related quality of life in patients with endometriosis: study protocol for a randomized controlled trial

**DOI:** 10.1186/s13063-022-06204-0

**Published:** 2022-04-12

**Authors:** Kathrin Schubert, Johanna Lohse, Matthias Kalder, Volker Ziller, Cornelia Weise

**Affiliations:** 1 Dept. of Psychology, Division of Clinical Psychology and Psychotherapy, Philipps-University Marburg, Marburg, Germany; 2 Clinic for Gynecology and Obstetrics, University Hospital of Giessen and Marburg (UKGM), Marburg, Germany

**Keywords:** CBT, Endometriosis, Women’s health, RCT, Chronic pelvic pain, Internet-based intervention

## Abstract

**Background:**

The trial investigates the efficacy of internet-based cognitive behavioral therapy (iCBT) in improving health-related QoL in patients with endometriosis, which is a chronic gynecological condition affecting up to 15% of people with female-assigned reproductive organs. Endometriosis is stress-related and comes with various physical symptoms such as pelvic pain and infertility. It has a substantial impact on health-related quality of life (QoL), and mind-body interventions seem promising in reducing the psychological burden.

**Methods:**

This is a monocentric randomized-controlled trial recruiting 120 patients with endometriosis. The intervention consists of eight iCBT modules focusing on psychoeducation, cognitive restructuring, pacing, and emotion regulation. Participants will receive written feedback from a trained therapist weekly. The comparator is a waitlist control group. All participants will be followed up 3 months after the intervention, and the intervention group will additionally be followed up 12 months after the intervention. Trial participants will not be blinded to the allocated trial arm. Primary outcome measures are endometriosis-related QoL, pain, and pain-related disability. Secondary outcomes include coping, illness representations, and psychological flexibility. Statistical analyses will be performed following intention-to-treat principles.

**Discussion:**

This randomized-controlled trial is the first trial to test the efficacy of iCBT for improving endometriosis-related QoL. Potential predictor variables and key mechanisms in treatment will be investigated to enable further progression in medical and psychological care for patients with endometriosis.

**Trial registration:**

ClinicalTrials.gov, NCT05098444 Registered on October 28, 2021

## Administrative information

Note: the numbers in curly brackets in this protocol refer to SPIRIT checklist item numbers. The order of the items has been modified to group similar items (see http://www.equator-network.org/reporting-guidelines/spirit-2013-statement-defining-standard-protocol-items-for-clinical-trials/).

**Table:** 

Title {1}	Internet-based Cognitive Behavioral Therapy for Improving Health-Related Quality of Life in Patients with Endometriosis: Study Protocol for a Randomized Controlled Trial.
Trial registration {2a and 2b}.	ClinicalTrials.gov, Identifier NCT05098444. Date of registration: 10/28/2021
Protocol version {3}	First version (dated 11/11/2021)
Funding {4}	Philipps-University Marburg, Marburg, Germany. This trial is non-commercial. We received a local grant called *UMRvernetzt* from Philipps-University Marburg, which is set up to encourage interdisciplinary exchange. The grant went to the construction of the iCBT platform. There is no external funding for this study.
Author details {5a}	^1, 2, 5^ Philipps-University Marburg, Dept. of Psychology, Division of Clinical Psychology and Psychotherapy, Marburg, Germany ^3, 4^ Clinic for Gynecology and Obstetrics, University Hospital of Giessen and Marburg (UKGM), Marburg, Germany.
Name and contact information for the trial sponsor {5b}	Trial Sponsor: UMR 2027, Philipps-University Marburg. Address: UMR 2027, Biegenstraße 36, 35032 Marburg, Germany. Email: interaktion@uni-marburg.de
Role of sponsor {5c}	The funder has no role in study design, analysis, interpretation, or publication of the study protocol and trial results.

## Introduction

### Background and rationale {6a}

#### Background

Endometriosis, defined by the presence of endometrial-like tissue outside the uterine cavity, is an inflammatory, chronic disease often associated with pelvic pain [1], infertility [2], and reduced quality of life (QoL) [3]. Prevalence of 5% up to 15% in women of reproductive age has been reported [4–6]. The societal and economic burden of endometriosis is severe, with high costs for medical treatment, reduced productivity at work [7], and high rates of absence from work [8]. The diagnosis of endometriosis is obtained only through laparoscopic surgery and histological confirmation. Due to a wide array of symptoms (both cyclic and acyclic [9]) and lack of awareness [10], practitioners often experience difficulties in diagnosing endometriosis correctly and in a timely manner [11]. This can lead to diagnostic delays of up to 10 years [12, 13] and prevents patients from receiving adequate treatment, adding further distress to the experienced psychological burden. Endometriosis is associated with symptoms of depression, anxiety [14, 15], and heightened feelings of uncertainty [16], leading to substantial reduction [3] in health-related quality of life (HRQoL). Dyspareunia, infertility, or decreased general ability to cope with everyday life [17] might put a strain especially on intimate relationships [18], impairing relationship satisfaction [19], and adding to the social burden of endometriosis [20]. The presentation of physical symptoms seems consistent across populations with different cultural backgrounds and geographical locations [7, 21]. However, HRQoL might be further reduced by culturally specific societal expectations, with, for example, women of Arab ancestry in the Middle Eastern region experiencing greater distress when suffering from infertility [22].

The exact etiology and pathogenesis of endometriosis remain unclear, although some biological and psychosomatic mechanisms and circuits involved in the progression of endometriosis have been identified: endometriosis is stress-related, with symptoms of endometriosis causing an increase in perceived psychological stress [23, 24] as well as high levels of chronic stress promoting overregulation of the hypothalamic–pituitary–adrenal (HPA) axis [25], leading to chronic inflammatory dysregulation and further growth of endometriotic lesions [26]. Chronic inflammation contributes to enhanced sensibility of nociceptive afferents [27], supporting peripheral and central sensitization processes [28] in regard to chronic pelvic pain (CPP) as one of the cardinal symptoms [1]. Overall, the complex pathogenesis might contribute to the existing knowledge gap regarding the precise mental health sequelae of endometriosis and unsubstantiated presumptions about the causality between physical and psychological symptoms [29].

There is no remedial treatment for patients with endometriosis available: it is a chronic disease with high recurrence rates after surgical or pharmacological treatment. Medical treatment of endometriosis is symptom-based, often focused on pain relief and treating infertility [30]. Surgical treatment is directed at ablating or excising endometriotic tissue. Pharmacological treatment is often directed at either symptomatic relief of pain through the use of analgesics (e.g., non-steroidal anti-inflammatory drugs) or at reducing circulating estrogen to a constantly lower level [31], leading to reduced ovarian function, less pain and inhibition in further growth of endometriotic tissue [32].

Given the high impact of endometriosis on HRQoL and the immediate influence of high psychological distress on its pathogenesis, developing complementary, non-medical treatment options for stress reduction and better psychological coping seems sensible. Diverse approaches have been tested over the last few decades. Different mind-body interventions for improving HRQoL in patients with endometriosis have been proven effective so far, including yoga [33], progressive muscle relaxation [33], and Traditional Chinese Medicine–based acupuncture combined with psychological consultations [35]. Kold, Hansen, Vedsted-Hansen, and Forman [36] conducted a prospective pilot single-arm trial, showing that mindfulness-based psychotherapy can lastingly improve HRQoL in patients with endometriosis. Yet overall, few studies have examined the effects of psychological and mind-body-interventions, most of them with limiting conclusions about efficacy due to rather low methodological quality (see [37, 38] for an overview).

#### Study rationale

There is an ongoing need for an evaluated, standardized, effective intervention for improving QoL in patients with endometriosis [39], although the exact interaction between physical and psychological symptoms is still to be further investigated. CBT teaches restructuring of unhelpful thoughts and core beliefs as well as behavioral activation and relaxation, leading to greater self-efficacy and enabling patients to display improved health behavior [40]. There is strong evidence for the effectiveness of CBT in treatment of chronic pain [41] and for stress reduction [42]. With perceived stress perpetuating experienced mental health sequelae and CPP as a cardinal symptom, we believe that the implementation CBT might be of great value for patients with endometriosis. Its efficacy in easing the burden of other disorders in women’s health (e.g., premenstrual dysphoric disorder [PMDD] [43]) further supports the implementation of CBT in treatment of endometriosis. Additionally, patients with endometriosis supported the implementation of CBT when informed about possible benefits [44]. However, to our knowledge, no study has yet investigated the effect of CBT in improving HRQoL in patients with endometriosis. We want to address this gap by testing the efficacy of CBT in improving HRQoL and examining potential moderators for new insights on key mechanisms in treatment.

An internet-based approach offers better accessibility alongside with reduced stigma associated with seeing a psychologist. Especially in patients with endometriosis, fear of not being taken seriously by a medical provider, fear of stigmatization, or disappointment in former practitioner-patient-relationships might otherwise hinder them from seeking psychological treatment [45]. We therefore decided to develop an internet-based CBT (iCBT) program. Such programs have previously been proven effective in the treatment of psychological [46] as well as psychosomatic [47] and somatopsychic [48] disorders and are considered as effective as face-to-face-therapy [49, 50]. We hypothesize that endometriosis-related QoL will be significantly improved when patients take part in our iCBT training.

### Objectives {7}

The trial objectives are:

- To test the efficacy of iCBT in improving HRQoL in patients with endometriosis
- To investigate potential predictor and mediator/moderator variables of treatment outcomes
- To improve medical and psychological care for patients with endometriosis, whose symptoms are often dismissed by medical providers
- To support further development of research within the field of women’s mental health with a clear focus on little-researched disorders relevant to people with female-assigned reproductive systems
- To foster the availability of online-interventions as a way of reducing barriers to appropriate care for patients with stigmatized diagnoses

### Trial design {8}

This is a randomized-controlled, two-armed, superiority trial testing the efficacy of an 8-week iCBT training in improving HRQoL in patients with endometriosis against a waitlist control group. Randomization will be balanced with a 1:1 ratio for patient allocation to the intervention group and waitlist control group. Identifying potential moderators and mediators of treatment outcome will also be a component of this trial.

## Methods: participants, interventions, and outcomes

### Study setting {9}

Patients with endometriosis will be recruited in Germany, and participants will access iCBT using their own technical devices (e.g., notebook, PC).

### Eligibility criteria {10}

The inclusion criteria are:

- Endometriosis diagnosed through laparoscopic surgery
- Age between 18 and 45
- Reduced HRQoL assessed with EHP-30 + 23 [52]
- Sufficient German language skills
- Access to an appropriate technical device (PC, laptop) with a stable internet connection

The exclusion criteria are:

- Current diagnosis of one of the following mental illnesses: unipolar severe depression, acute suicidal tendencies, bipolar affective disorder, unipolar manic episodes, psychotic disorder, alcohol or substance use disorder (assessed using the Web-Based Screening Questionnaire (WSQ) [53], and Beck Depression Inventory–II (BDI-II) [54]
- Receiving CBT treatment, current or in the past 2 years
- Regular intake of benzodiazepines, changes in intake (e.g., start/change in dosage/discontinuation) of antidepressants or hormonal contraceptives within the last 3 months
- Current or planned IUI, IVF, or ICSI treatment with hormonal stimulation within the next 8 months
- Current pregnancy or birth of a child within the last 6 months, breastfeeding within the last 6 months
- Current diagnosis of one of the following: malignant tumor, esp. breast cancer, cervical cancer, or ovarian cancer; ulcerative colitis; Crohn’s disease; or any bacterial or viral infection such as TC, HAV, HBV, or HCV

### Who will take informed consent? {26a}

Informed consent will be obtained through local researchers experienced in working with patients with endometriosis. Potential trial participants receive detailed written information about trial scope, time schedule, randomization process, and so forth. During an extensive discussion of general trial benefits and risks, as well as use of trial participation via telephone, potential participants will be given the chance to address individual questions and concerns. Afterwards, participants will be asked to send written informed consent within the next few days to ensure that consent is given voluntarily without situational pressure.

### Additional consent provisions for collection and use of participant data and biological specimens {26b}

After trial completion, participants will be asked if they agree to be contacted in the future about further research participation. No collection of biological specimens is planned.

### Interventions

#### Explanation for the choice of comparators {6b}

The comparator is a waitlist control. Patients in the waitlist control group can continue with their usual treatment plan during trial participation (e.g., medication or physiotherapy). Because there is, as yet, no established treatment as usual (TAU) for patients with endometriosis, using a waitlist control seemed reasonable to capture real-life conditions. Patients currently receiving psychotherapy or seeking psychotherapeutic treatment in the past 2 years will be excluded from trial participation.

#### Intervention description {11a}

The online intervention lasts 8 weeks with one assigned interactive module per week, each of which consists of psychoeducational information, exercises, and homework. Different formats, methods, and media (e.g., infographics, videos, audio files, and text) will be used to convey relevant knowledge and strategies. For greater vividness, four fictional personas are introduced in the beginning; their answers and thoughts on certain exercises are displayed as encouraging examples. The intervention content was developed with helpful support from researchers with different scientific backgrounds and expertise in psychology, medicine, and gender studies. Additionally, the first three modules were tested by five endometriosis patients interested in iCBT. They were interviewed about their experience and provided valuable feedback on usability, psychoeducational comprehensiveness, and overall benefit.

Participants generate an individual biopsychosocial model about the impact of endometriosis on different areas of life and get to learn about cognitive restructuring. Relations between stress and pain are explored, and participants are encouraged to try out different pacing and relaxation methods. Acceptance and regulation of difficult emotions are practiced, as well as communication of healthy boundaries in different social settings. The last module focuses on reflection and long-term maintenance of achieved progression. Table 1 describes the intervention content in detail.

**Table 1:** Content of the different modules

Week	Module name	Module content
1	**Module 1:** Getting started with the training	Psychoeducation, goal setting
2	**Module 2:** Thoughts I—Our constant companions	Cognitive restructuring I—Using the ABC model
3	**Module 3:** Thoughts II—Who controls whom?	Cognitive restructuring II—Using the ABCDE model
4	**Module 4:** My pain and me—And still I want to do something	Dealing with acute pain, pacing, weekly schedule
5	**Module 5:** Stress and pain—Always everything at once	Stress management (recovery skills, dealing with acute stress, contingency plans)
6	**Module 6:** Emotions—Accepting and tolerating	Psychoeducation, accepting and regulating negative emotions, mindfulness
7	**Module 7:** Clear communication	Basic communication rules, flexible handling of different situations
8	**Module 8:** My plan for the future	Summary, setting of long-term goals

Participants are asked to spend 1 to 2 h on each module. After each week, participants receive written feedback from a trained psychologist based on their input into the treatment platform. The participants can ask questions regarding the module they have been working on via text messages to their psychologist on the online platform. The psychologist is at least a bachelor-level clinical psychologist and receives regular supervision by a licensed CBT therapist.

#### Criteria for discontinuing or modifying allocated interventions {11b}

There are no criteria for discontinuing or modifying the intervention. Participants may withdraw from voluntary trial participation at any point for any given reason. Withdrawal from participation has no negative consequences or disadvantages for the participant. The investigators might also terminate participation if severe adverse events arise during the intervention.

#### Strategies to improve adherence to interventions {11c}

Adherence (e.g., taking part in the online intervention weekly) is encouraged through weekly contact with trained psychologists; strategies may include encouragement as well as setting specific goals and a specific date for working on the weekly modules or for certain exercises.

#### Relevant concomitant care permitted or prohibited during the trial {11d}

Any treatment or change in treatment with direct influence on trial outcomes is permitted during the trial as defined in the exclusion criteria (see {10}).

#### Provisions for post-trial care {30}

No special provisions are offered.

### Outcomes {12}

#### Primary outcome measures

The primary outcomes are improvements in endometrioses-related QoL and pain during menstruation and across the cycle, as well as pain-related disability in the time between baseline and follow-up. Greater improvements are expected in the intervention group compared to the control group. Endometriosis-related QoL will be assessed using the Endometriosis Health Profile-30 + 23 (EHP-30+23) [55], which measures endometriosis-related burden across different domains of everyday life considered relevant to patients with endometriosis. The core questionnaire of 30 items is complemented by a 23-item modular questionnaire with six subscales (work life, relationship with children, sexual intercourse, medical profession, treatment, and infertility). Items are rated on a Likert scale ranging from 0 to 4. A score ranging from 0 to 100 is calculated for the core questionnaire as well as for each modular subscale, with 0 indicating the best possible quality of life.

Pain will be measured using visual analog scales. Pain-related disability will be assessed using the Pain Disability Index (PDI) [56], a 7-item questionnaire measuring the degree of disruption in different aspects of everyday life caused by chronic pain (e.g., family/home responsibilities, recreation). Each item is rated on an 11-point Likert Scale (0–10). Raw scores for all items are added together, with higher scores indicating higher degrees of disruption caused by chronic pain. Primary outcomes will be assessed at baseline, post-treatment, and at 3 months past treatment, as well as at 12 months past treatment for the intervention group.

#### Secondary outcome measures

All secondary outcome measures will be assessed at the same points of measurement as the primary outcome measures.

#### Depressive mood

Endometriosis is often associated with a wide array of depressive symptoms. Depressive mood is assessed using the Patient Health Questionnaire-9 (PHQ-9) [57], a 9-item depression subscale from the Patient Health Questionnaire which has reliable sensitivity to change.

#### Perceived stress

As described in {6a}, endometriosis and its impact on HRQoL are closely linked to perceived stress. The perception of psychological stress (e.g., the extent to which participants experience themselves not being able to cope with their situation) will be measured using the Perceived Stress Scale-10 (PSS-10) [58].

#### Coping ability

General abilities in coping with difficulties and challenging situations play an important role in adapting to life with a chronic illness. The improvement of adaptive coping skills can reduce perceived stress [59]. The Brief COPE [60] is a 28-item measurement that captures various facets of coping using the subscales of problem-focused, emotion-focused, and avoidant coping.

#### Cognitive illness representations

Individuals suffering from chronic illness develop a set of cognitive representations about their disease, its cause and impact, and their ability to control progression. These representations form health-related behavior and are assessed using the Illness Perception Questionnaire Revised (IPQ-R) [61].

#### Psychological flexibility

The construct of psychological flexibility derives from research about therapeutic processes in Acceptance and Commitment Therapy [62]. Although CBT is tested in this trial, a shift from experiential avoidance of negative inner experience (e.g., unpleasant thoughts and emotions) toward a more accepting approach seems helpful in living with a chronic illness. Therefore, psychological flexibility will be captured with the 7-item Acceptance and Action Questionnaire-II (AAQ-II) [63].

#### Control variables

The control variables will be assessed at baseline only.

#### Relationship satisfaction

Living with endometriosis can have a negative impact on relations with others, and especially on committed relationships with romantic partners. Endometriosis leaves couples to deal with many different difficulties such as reduced ability to cope with stressors, infertility, and sexual distress [64]. At the same time, a functional, healthy relationship can be a source of continuous support and may improve resilience in living with the disease [44]. Relationship satisfaction will be assessed using the 7-item Relationship Assessment Scale (RAS) [65].

#### Personality traits

To determine the role of personality traits as potential moderators of treatment outcome, the Big Five Inventory-10 (BFI-10) [66] will be used to capture extraversion, agreeableness, conscientiousness, emotional stability, and openness.

#### Process evaluation

To evaluate the planned CBT treatment and for identification of elements to focus on for further improvement, the participants are asked to provide weekly feedback on the module. We will therefore monitor symptoms and impact on HRQoL weekly throughout the training and present the questionnaire alongside a section for further comments on the module and aspects that have been helpful or unnecessary for each participant. Negative effects of treatment will be measured post-treatment and at follow-up.

#### Endometriosis-related quality of life

For a quick assessment of HRQoL, the Endometriosis Health Profile-5 (EHP-5) [67] will be used weekly after each module. This 5-item short form of the EHP-30 + 23 provides sufficient reliability and validity [67].

#### Negative effects of treatment

To detect possible negative effects of treatment in this trial (e.g., increased stress, new symptoms, hopelessness, and dependency), the Negative Effect Questionnaire (NEQ) [68] will be implemented.

### Participant timeline {13}

**Fig. 1:**
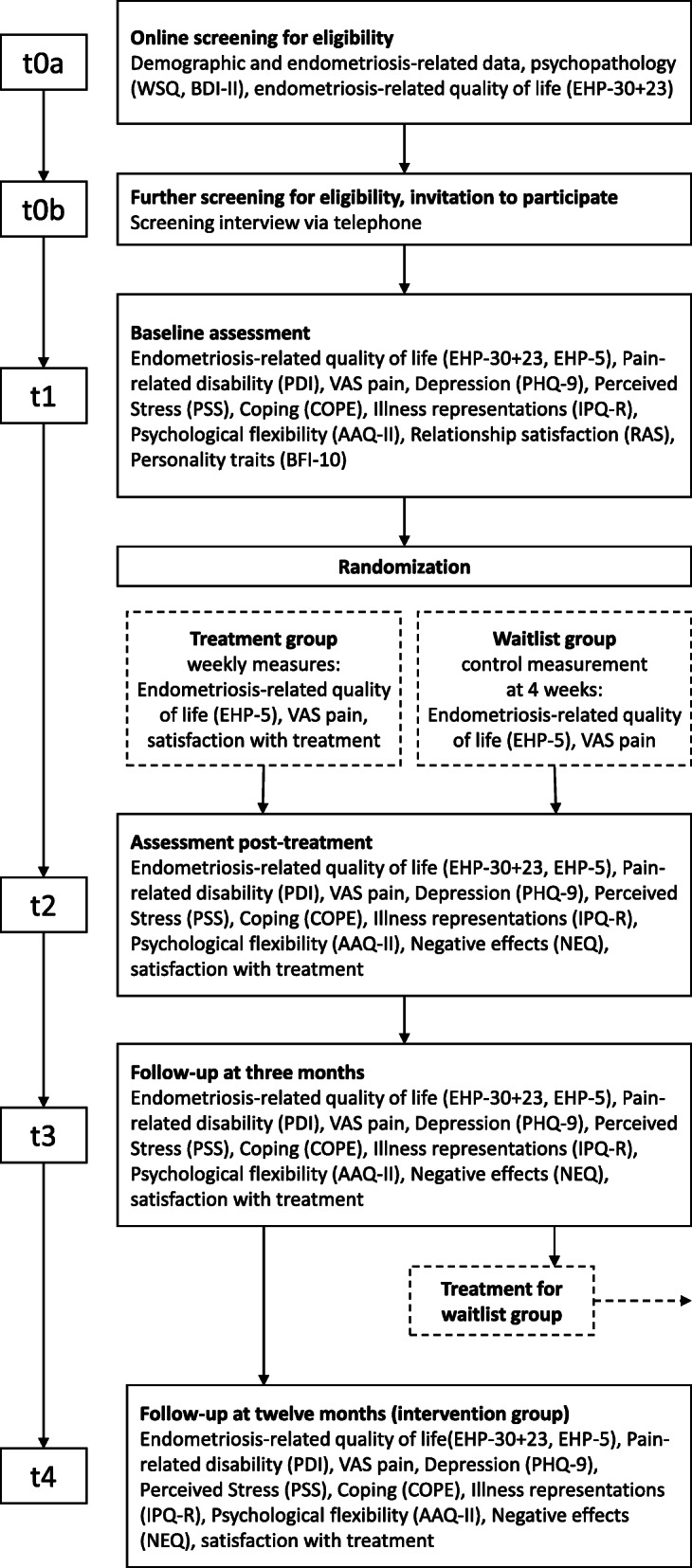
Participant timeline

For the participant timeline, see Fig. 1.

#### Sample size {14}

Because this is the first published study to test the efficacy of iCBT for patients with endometriosis, there is not much data available to guide sample size calculations. We estimated an effect size of *f* = 0.25, drawing on another trial in women’s health that tested efficacy of iCBT in PMDD [42]. A total number of 120 participants are needed with significance of *p* < .05 and 95% power.

#### Recruitment {15}

Participants will be recruited online via social media and mailing lists, as well as through local inpatient clinics and gynecologists. Patients interested in participation will receive a link to the online screening for our trial.

### Assignment of interventions: allocation

#### Sequence generation {16a}

Participants will be randomized to either the treatment or the control group using blockwise randomization. This is to ensure balanced group size even when the target sample size cannot be reached. Block size will not be shared with researchers running the trial.

#### Concealment mechanism {16b}

A web-based randomization system will be used to implement the planned allocation sequence.

#### Implementation {16c}

Randomization will be implemented by a research assistant who is not involved in the trial in any form. Participants will be informed about their allocation via mail.

### Assignment of interventions: blinding

#### Who will be blinded {17a}

Due to the nature of the planned intervention and trial design, researchers and participants will not be blinded to the allocated trial arm.

#### Procedure for unblinding if needed {17b}

There is no need for any kind of unblinding procedure, as no one involved is blinded.

### Data collection and management

#### Plans for assessment and collection of outcomes {18a}

Data will be assessed online via self-report measures. Primary and secondary outcomes will be assessed at baseline, post-treatment, at a 3-month follow-up and, for the intervention group, at a 12-month follow-up. Control variables will be assessed at baseline only; measures for process evaluation will be assessed weekly during treatment and at post treatment as well as follow-up. For more details on outcome measures see {12}.

#### Plans to promote participant retention and complete follow-up {18b}

Participants will be contacted for follow-up via e-mail. The data are collected online, reducing as many barriers as possible (e.g., having to schedule an in-person appointment or sending the questionnaires back). There is no additional incentive for participants to complete the trial. Based on our experience working with patients who suffer from endometriosis, the sample is usually grateful for any research conducted on endometriosis and tends to be very eager to participate.

#### Data management {19}

Every participant will get an individual trial subject number used to identify them throughout the trial. Researchers involved in the trial will monitor data entry and check for any missing data or anomalies.

#### Confidentiality {27}

All data will be stored on local servers of the Philipps-University Marburg according to GDPR and local policy. All data, including collected personal information, will be kept strictly confidential.

#### Plans for collection, laboratory evaluation, and storage of biological specimens for genetic or molecular analysis in this trial/future use {33}

There are no plans for collection and evaluation of biological specimens.

### Statistical methods

#### Statistical methods for primary and secondary outcomes {20a}

Analysis will be conducted based on the intention-to-treat (ITT) principle, and two-sided *p*-values < .05 will be considered statistically significant. Results will be reported according to the Consolidated Standards of Reporting Trials (CONSORT, [53]). Baseline data will be summarized by treatment group and total. We will report categorical data as *N* (%), continuous data as mean (SD). Primary outcome analysis will be performed as a general linear model comparing the difference in mean EHP-30 + 23 score and pain scores between groups with repeated measures (baseline, post-treatment, 3-months follow-up). With this approach, all participants providing data for at least one point of measurement will be included. The Holm-Bonferroni Method [54] will be implemented to account for the testing of multiple hypotheses due to nomination of two primary outcomes. For secondary outcomes, we will use analysis of covariance models to conduct possibly necessary adjustment for baseline characteristics. If any non-foreseeable changes occur during the trial, we will adjust the statistical analysis plan accordingly. Sensitivity analyses will be performed to investigate the possible influence of missing data on the robustness of the results.

#### Interim analyses {21b}

No interim analyses are planned.

### Methods for additional analyses (e.g., subgroup analyses) {20b}

#### Subgroup analyses

We will test for possible subgroup factors such as being partnered and currently trying to conceive. These are known moderators for HRQoL reductions through endometriosis. As our sample is expected to be quite homogenous in age and gender, other subgroup analyses might not be functional.

#### Process evaluation

To evaluate the development of iCBT for patients with endometriosis, the weekly measures of endometriosis-related QoL and qualitative feedback are taken into account. We hope to identify helpful aspects and key mechanisms in improving HRQoL to further improve the intervention. No systematic qualitative approach in assessing participant feedback will be implemented.

### Methods in analysis to handle protocol non-adherence and any statistical methods to handle missing data {20c}

Data will be analyzed following the ITT principle. Missing data will be explored. Imputation of missing values is not intended, although multiple imputations might be used within sensitivity analyses assuming data are missing at random.

### Plans to give access to the full protocol, participant level-data, and statistical code {31c}

There is no additional protocol to this version. Because gynecological diseases still carry some stigma, we will decide on whether to publish participant-level data anonymously after trial completion. Psychological treatment of endometriosis is not yet common, so participants might easily be identified through some of the collected data. Only if we reach a sample size large enough to rule out identification of participants by some of their data (*N* ≥ 80) will we proceed to provide access to participant-level data without any kind of demographic information. Any other data, statistical code, or documentation may be provided by the corresponding author at request.

### Oversight and monitoring

#### Composition of the coordinating center and trial steering committee {5d}

The immediate trial team responsible for trial management, day-to-day support, and administration of the intervention will meet weekly. The wider team—including medical experts—will meet every 3 months. Due to the small size of our trial and the few researchers being involved, other committees such as a Trial Steering Committee or Project Management Group will not be formed.

#### Composition of the data monitoring committee, its role and reporting structure {21a}

Given the small size of our monocentric trial with few researchers involved and without external funding, there is no need for an additional data monitoring committee.

#### Adverse event reporting and harms {22}

Adverse events (AEs) will be collected and monitored by the therapists running the intervention. Any disadvantageous event occurring during the trial (e.g., worsening of symptoms, acute suicidal ideation, hospitalization due to mood deterioration) will be classified as an adverse event. Both AEs that are assumed to be a consequence of treatment and those that are not causally related to the treatment will be collected. All AEs will be discussed weekly regarding causality and severity, and possible further actions will be considered.

#### Frequency and plans for auditing trial conduct {23}

The trial will be monitored by the principal investigator (CW) and the team members KS and JL. There will be no additional independent auditing of trial conduct.

#### Plans for communicating important protocol amendments to relevant parties (e.g. trial participants, ethical committees) {25}

The local ethic committee will be notified for approval if any protocol modifications are required. Trial registries and the trial protocol would be updated.

### Dissemination plans {31a}

Trial results will be communicated via journal publications and conference presentations. If participants state their interest in trial results, they will be informed after trial completion.

## Discussion

This trial is designed to test the efficacy of iCBT in improving HRQoL in patients with endometriosis. Endometriosis is a chronic disease linked to symptoms of stress, depression, and anxiety. There is a need for additional, non-medical interventions targeting mental health and reducing the burden of living with endometriosis [55]. The intervention is investigated using a waitlist control as the comparator.

### Limitations

The decision to use waitlist control in our study comes with the risk of possible overestimation of treatment effects [56]. There is no TAU established for treatment of endometriosis, so using a TAU control group was not considered. Because it seemed ethical not to withhold a potential effective treatment for the control group, including a simple control group or placebo control group was also ruled out. We will investigate key mechanisms in improvement of HRQoL, allowing for the development of potential active control groups in future trials. The online setting of our invention limits access for patients without a suitable electronic device and/or skills needed to participate in iCBT (e.g., basic computer skills); however, if iCBT is proven effective in reducing the burden of living with endometriosis in this trial, the intervention can easily be adapted to face-to-face therapy for further research.

### Strengths

Research about mind-body interventions for improvement of HRQoL in patients with endometriosis is still limited, and the demand for more RCTs has been mentioned [36, 37]. To our knowledge, this is the first RCT testing the efficacy of iCBT for patients with endometriosis in a cost-efficient way. The study offers iCBT as an established mind-body intervention to patients with a diagnosis that comes with an array of symptoms and who often are not taken seriously by both practitioners and in their private lives. Our ultimate goal is to discover effective support for patients with endometriosis and to encourage further research within this domain.

## Trial status

The trial is registered (dated October 28, 2021) at ClinicalTrials.gov, Identifier NCT05098444, recruitment will start in January 2022. The current protocol is version 1 (dated November 11, 2021).

## Abbreviations

AAQ-II: Acceptance and Action Questionnaire II
AE: Adverse event
BDI-II: Beck’s Depression Inventory II
BFI-10: Big Five Inventory 10
CBT: Cognitive behavioral therapy
CONSORT: Consolidated Standards of Reporting Trials
CPP: Chronic pelvic pain
EHP-30 + 23: Endometriosis Health Profile 30 + 23
EHP-5: Endometriosis Health Profile 5
GDPR: General Data Protection Regulation
HAV: Hepatitis A virus
HBV: Hepatitis B virus
HCV: Hepatitis C virus
HRQoL: Health-related quality of life
iCBT: Internet-based cognitive behavioral Therapy
ICSI: Intracytoplasmic sperm injection
IPQ-R: Illness Perceptions Questionnaire Revised
ITT: Intention-to-treat principle
IUI: Intrauterine insemination
IVF: In vitro fertilization
NEQ: Negative Effects Questionnaire
PC: Personal computer
PDI: Pain Disability Index
PMDD: Premenstrual dysphoric disorder
PSS-10: Perceived Stress Scale 10
QoL: Quality of life
RAS: Relationship Assessment Scale
SD: Standard derivation
TAU: Treatment as usual
TBC: Tuberculosis
TCM: Traditional Chinese Medicine
WSQ: Web-Based Screening Questionnaire
